# Migration and Accumulation of Uranium-Associated Heavy Metals in Mining-Affected Ecosystems (Water, Soil, and Plants)

**DOI:** 10.3390/biology15060502

**Published:** 2026-03-20

**Authors:** Madina Kairullova, Meirat Bakhtin, Kuralay Ilbekova, Danara Ibrayeva

**Affiliations:** Science Research Institute of Radiobiology and Radiation Protection, Astana Medical University NJSC, Astana 010000, Kazakhstan; bakhtin.m@amu.kz (M.B.); ilbekova.kb@gmail.com (K.I.)

**Keywords:** uranium mining, heavy metals, soil contamination, water quality, bioaccumulation, vegetation, ecological risk, health risk assessment

## Abstract

Uranium mining generates complex multi-element contamination that affects interconnected ecosystem components, posing long-term ecological and sanitary risks; this review places these impacts in a broad environmental context and aims to synthesize current knowledge on the distribution, migration, and accumulation of uranium and associated heavy metals in water, soil, and plants. A structured analysis of international peer-reviewed literature was conducted, focusing on documented pathways of metal release from tailings and waste dumps, geochemical controls on mobility, and biological uptake by vegetation. The reviewed studies consistently show that tailings and disturbed ore-bearing strata act as persistent sources of uranium and heavy metals (e.g., Cd, Pb, Cr, Ni, Zn, Mn, As), which migrate through infiltration, acid mine drainage, and atmospheric dispersion, leading to elevated concentrations in surface and groundwater and long-term accumulation in soils. Soils function as the principal sink controlling metal bioavailability, while vegetation reflects the bioavailable fraction and exhibits pronounced species-specific accumulation patterns. These processes establish an active “soil–water–plant” transfer chain that facilitates entry of contaminants into food webs. The synthesis indicates that combined uranium and heavy metal contamination represents a sustained ecological and public health concern in uranium-mining regions and underscores the need for integrated monitoring of soils, waters, and vegetation, along with quantitative risk assessment and scientifically grounded remediation strategies.

## Simple Summary

Uranium mining provides important raw materials for energy production, but it can also leave harmful substances in the environment long after mining stops. These substances include uranium and other toxic metals that can spread into water, soil, and plants. This study reviews scientific research to understand how these pollutants move through nature and where they accumulate. The results show that mining waste, such as tailings and waste rock, acts as a long-term source of contamination. Pollutants can be carried by rainwater into rivers and underground water, stored in soils for many years, and taken up by plants growing in polluted areas. Some plants accumulate these substances in their leaves and stems, allowing the pollutants to enter food chains and eventually affect people and animals. The study concludes that pollution from uranium mining is not confined to a single part of the environment but spreads through interconnected systems of water, soil, and living organisms. Understanding these pathways is important for protecting ecosystems and human health. The findings are valuable for improving environmental monitoring, guiding cleanup actions, and helping communities and decision-makers reduce the risks posed by contaminated land and water.

## 1. Introduction

Human activities have intensified their impacts on natural ecosystems, raising serious concerns about the environmental conditions in uranium mining regions worldwide [1]. Uranium extraction methods, such as open-pit mining, heap leaching, and in situ leaching, disturb land and mobilize radionuclides and toxic metals, redistributing them across various environmental compartments [2,3]. Factors like precipitation, weathering, and leaks from tailings or waste storage facilities facilitate the migration of these contaminants beyond mining sites, integrating them into natural biogeochemical cycles and forming secondary pollution zones [4,5]. For example, uranium mill tailings (UMT) are a major concern due to high levels of radioactive and toxic elements, including uranium, thorium, and lead, which pose serious risks to terrestrial and aquatic systems and human health [4]. The leaching behavior of U, ^226^Ra, and ^210^Pb from uranium tailings is significantly influenced by pH, demonstrating potential radioactive contamination of surrounding environments [6].

Kazakhstan holds a prominent position in the global uranium industry, accounting for approximately 14% of the world’s reserves. Its annual production exceeds 23,000 tonnes, making Kazakhstan a key participant in the global nuclear fuel cycle [7]. While uranium mining brings significant economic and strategic benefits, it also poses long-term environmental risks, particularly soil and water contamination.

Uranium is a unique pollutant that exhibits both radiological and chemical toxicity. Mining activities are rarely limited to uranium alone; they are often accompanied by the release of other heavy metals such as Cd, Cu, Pb, Mn, and Zn. These elements accumulate in soils, surface and groundwater, and sediments, generating complex multi-element geochemical anomalies and substantially altering ecosystem functioning [8,9,10,11].

Heavy metals are characterized by high persistence, pronounced bioaccumulation, and the ability to migrate through ecological pathways. Even at low concentrations, they can exert chronic and cumulative toxic effects on soil microorganisms, vegetation, aquatic organisms, and ultimately human health through inhalation, dermal contact, or dietary intake. Vegetation plays a critical dual role in this context: it serves as a sensitive bioindicator of contamination and as a key vector for transferring toxic elements through food webs [12,13,14].

Research indicates that the ecological consequences of uranium mining persist long after operations cease. For example, at the former Korday uranium mine in Kazakhstan, elevated concentrations of uranium and associated heavy metals (As and Mn) have been detected in local water bodies, demonstrating the long-term persistence of mining-related pollution [15]. Similarly, in the vicinity of the decommissioned Grachevskoye deposit, significant increases in uranium and heavy metal concentrations (Fe, Zn, Ba) were recorded in surface and groundwater in the Saumalkol settlement between 2005 and 2024, reflecting prolonged hydrogeochemical impacts from abandoned sites [16]. Studies focusing on water contamination in settlements near uranium mining territories in Northern Kazakhstan confirm heightened risks of metal and radionuclide contamination in groundwater resources [17,18].

In China, uranium, thorium, cadmium, and chromium concentrations in surface waters and sediments near the Xiangshan uranium deposit (Jiangxi Province) were found to far exceed background levels. Elevated pollution indices (CF, Igeo, RI) indicate high ecological risks to aquatic ecosystems, highlighting uranium mining’s ability to generate persistent, multi-element anomalies and drive the long-term redistribution of toxic metals [19].

Despite Kazakhstan’s strategic role in uranium production, comprehensive ecological studies in its uranium-mining regions remain limited. Most research has focused on monitoring and statistical assessments, while systematic investigations into heavy metal migration, accumulation mechanisms, and ecological impacts are underdeveloped. In particular, the role of vegetation in the accumulation and transformation of heavy metals in uranium mining landscapes is still poorly understood.

This study aims to provide a structured, critical review of the scientific literature on heavy metal contamination in uranium-mining regions, emphasizing migration pathways, accumulation mechanisms, and ecological risks to key ecosystem components: water, soil, and vegetation. By synthesizing current knowledge, the review seeks to identify major scientific gaps and provide a foundation for evidence-based environmental risk assessment and management in uranium-producing areas.

## 2. Sources and Migration Routes of Heavy Metals in the Ecosystems of Uranium Mining Areas

Heavy metals are among the most significant anthropogenic pollutants impacting natural ecosystems. Their introduction into the environment is closely associated with the extraction and processing of mineral resources, especially polymetallic and uranium ores, as well as the transportation and technological handling of these materials. Since the Industrial Revolution, heavy metal emissions from human activities have increased dramatically, driven by the expansion of mining, metallurgy, and energy production. Additional sources, including domestic, industrial, agricultural, and transportation-related emissions and discharges, further contribute to the widespread dispersal of heavy metals into the atmosphere, soils, and water bodies.

Environmental contamination by heavy metals is complex and spatially uneven, reflecting the diversity of sources and local conditions. Major contributors include polymetallic ore mines and quarries, ferrous and non-ferrous metallurgical plants, metal-processing industries, coal-fired power stations, waste incineration facilities, and road traffic. Collectively, these activities give rise to persistent contamination hotspots in soils, surface and groundwater, and the atmosphere, exerting prolonged anthropogenic pressure on ecosystems [20].

Intensive economic activity results in substantial input of toxic metals into aquatic environments worldwide, primarily through industrial and agricultural effluents. Over time, heavy metals accumulate in sediments, microorganisms, aquatic plants, and fauna, triggering secondary contamination. Changes in physicochemical conditions during sedimentation, such as pH, redox potential (Eh), ionic composition, and microbial activity, can remobilize previously immobilized metals, reintroducing them into the water column and increasing risks to biota and human health via trophic transfer [21,22].

The ecological impact of heavy metal contamination is particularly severe in regions affected by uranium mining and processing. Here, anthropogenic pressures arise from a combination of aerosol emissions, the accumulation of waste rock and low-grade ore, hydrometallurgical tailings storage, and large volumes of liquid waste and slurries in tailings facilities. These technogenic sites act as long-term sources of both radionuclides and heavy metals, shaping a unique radioecological and ecotoxicological environment [23].

The migration of heavy metals within uranium-producing landscapes presents a significant environmental challenge, as mining activities introduce both radioactive and chemically toxic contaminants. This combination increases pollutant mobility and complicates the prediction of their behavior in the environment [24,25].

Once released from tailings, waste dumps, and other anthropogenic sources, heavy metals are mobilized by hydrological flow and geochemical processes and follow sequential migration pathways. They are first redistributed within soils, then transported to surface and groundwater systems, subsequently deposited in sediments, and ultimately taken up by vegetation and fauna. These processes involve bioaccumulation at the organism level and biomagnification along food chains, leading to ecosystem disruption and increased risks to human health [26,27,28].

Tailings facilities represent the main reservoirs of heavy metals in uranium mining regions, containing concentrated by-products of mining and processing operations [29,30]. These wastes are typically enriched not only in uranium but also in other toxic elements, such as As, Cd, Pb, and Tl, as well as additional hazardous components [25,27]. The release of metals from tailings and waste dumps is driven by natural processes, including leaching by precipitation, mechanical dispersion of fine particles, and atmospheric deposition. Metals subsequently enter local migration pathways, primarily within soils, where they accumulate, transform, and redistribute. Under certain geochemical conditions, some metals become more mobile, facilitating further transport via surface runoff and groundwater into rivers and aquifers [28].

Given the multi-stage nature of heavy metal migration and the intricate interplay of geochemical and biological processes, systematic mapping of the primary pathways of metal redistribution among environmental compartments is essential for ecological risk assessment. Table 1 presents the main migration routes of heavy metals in uranium mining regions, emphasizing dominant transport mechanisms, controlling factors, and potential ecological and health impacts.

Thus, the migration pathways summarized in Table 1 show that heavy metals released during uranium mining are redistributed among closely interconnected components of the environment. Soils represent the primary accumulation medium, while surface and groundwater play key roles in the transport and spatial redistribution of contaminants. Vegetation, in turn, serves as an important biological link, ensuring metal uptake and their subsequent entry into food chains. The interactions among these components largely determine metal mobility, bioavailability, and associated ecological and human health risks. In this context, the following sections focus on the characteristics of soil, water, and plant contamination as the principal elements of ecosystems in uranium mining regions.

## 3. Heavy Metals in Water

Mining in uranium-rich areas significantly affects the quality of both surface and groundwater. Water is highly susceptible to contamination by heavy metals. In aquatic environments, heavy metals may exist dissolved in water or attached to suspended particles that eventually settle to the bottom. Metals in dissolved form are more mobile and can be more easily absorbed by aquatic organisms. These metals interact with various components of water, such as organic matter, ions, microscopic algae, and bacteria.

Facilities that store large volumes of industrial solid waste, such as tailings storage sites and sludge disposal areas, play a major role in contaminating groundwater. Over time, these sites create surface areas that pose environmental risks. As precipitation seeps into the waste, contaminants, including heavy metals, nitrate and sulfate ions, and radionuclides, gradually migrate through the walls and bottoms of storage facilities. This process results in repeated infiltration of pollutants into the groundwater of upper aquifers [44].

Water migration is a fundamental process responsible for the long-distance transport of contaminants in both surface and groundwater environments. The primary mechanisms that initiate this migration are:

1. Infiltration of precipitation: Rainwater that seeps through waste rock piles and tailings mobilizes heavy metals, nitrate and sulfate ions, as well as radionuclides. These dissolved contaminants can move through the walls and bases of storage facilities, eventually reaching groundwater [45].
2. Acid mine drainage (AMD): The oxidation of sulfide minerals such as pyrite, which is commonly present in uranium ores and mining waste, produces acidic drainage with a low pH [46]. This process greatly increases the solubility and mobility of many metals, including uranium. In these conditions, uranium is oxidized to its more soluble hexavalent state (U(VI)), making it easier to transport into soils and river systems [46].

Studies have shown that contaminant migration can be significant. For instance, modeling of waste rock piles in the southern regions has revealed that heavy metals can migrate and affect groundwater for periods exceeding 1000 years [35]. The behavior of heavy metals in aquatic environments, including their mobility, transformation, and immobilization, is governed by a complex interplay of geochemical and biological factors [43]. In surface waters adjacent to uranium tailings, concentrations of certain heavy metals frequently surpass permissible thresholds by a considerable margin [47].

Investigations conducted in various uranium mining regions worldwide underscore serious environmental challenges. The extent of soil and water contamination may vary depending on local geological conditions and mining methods. Table 2 summarizes representative case studies from major uranium mining regions worldwide and compares heavy metal contamination in water.

The comparative analysis presented in Table 2 demonstrates that heavy metal contamination of water resources is a persistent and geographically widespread consequence of uranium mining. However, the dominant contaminants and their concentrations vary depending on geological settings and mining practices.

## 4. Heavy Metals in Soil

Soils represent the primary sink for heavy metals and radionuclides in uranium-mining regions and play a decisive role in the formation of environmental and public health risks. During uranium extraction and processing, the soil cover serves as the primary repository for contaminants originating from technogenic sources, such as mining operations, tailings storage facilities, and dust emissions, as well as from atmospheric deposition and water-mediated transport. This pattern has been consistently documented by numerous international studies conducted in areas surrounding both active and abandoned uranium mines in China, Morocco, and other countries [39,47,61,62,63].

Soils function as a long-term natural buffer controlling the redistribution of heavy metals among major components of ecosystems, including the atmosphere, hydrosphere, biota, and humans. Owing to the high sorption capacity of mineral and organic soil fractions, heavy metals can accumulate over decades or even centuries, forming persistent secondary contamination hotspots. Unlike air and water, where contaminant concentrations may decline relatively rapidly, the residence time of heavy metals in soils is considerably longer, making soil contamination a critical factor in long-term environmental risk assessment [64].

The accumulation of heavy metals distorts the natural geochemical signal of background element concentrations, including Ni, Cu, Zn, Mn, Cd, Co, and Pb, which under undisturbed conditions are largely controlled by the composition of parent materials. In technogenically altered landscapes of uranium-mining areas, a pronounced spatial heterogeneity in elemental distribution is observed, particularly for Pb, Cu, Cr, Ni, Zn, Mn, and Zr. These elements show a strong association with industrial activities and technological development, reflecting the intensity and legacy of anthropogenic impacts [65]. The most unevenly distributed elements in soils are Pb, Mo, Cu, Cr, Ni, Zn, Mn, and Zr, which are closely linked to modern technological processes and are among the most extensively used metals in industrial applications [66].

Of particular ecological relevance is the persistence of heavy metals within the soil profile. Even after the cessation of uranium mining and processing activities, soil contamination can remain for extended periods, as metal removal occurs slowly through processes such as leaching, biological uptake, erosion, and deflation [67]. The half-life times of heavy metals in soils vary widely and may range from several decades to millennia: 70 to 510 years for Zn, 13 to 110 years for Cd, 310 to 1500 years for Cu, and 740 to 5900 years for Pb [68,69].

International studies of uranium-mining areas consistently demonstrate a high degree of technogenic impact of heavy metals on soils. In zones adjacent to uranium mine tailings facilities, concentrations of Cd, Co, Cr, Cu, Mn, Ni, Pb, Zn, and U often substantially exceed local background levels [39,47]. For example, in the Xiaozhuang uranium mining district (China), soil concentrations of uranium and lead ranged from 4.1 to 206.9 mg/kg and from 43.3 to 126.0 mg/kg, respectively. The calculated geoaccumulation index (I_geo) values exceeded 1, indicating moderate to locally moderate–strong soil contamination [70].

Under conditions of elevated technogenic soil contamination, heavy metals are primarily incorporated into the biological cycle through vegetation, which serves as a key intermediary between the abiotic environment and biota. The uptake and accumulation of heavy metals by plants are associated with the development of physiological and morphological disturbances and create pathways for their subsequent transfer along trophic chains. At the same time, the accumulation of heavy metals in soils significantly influences soil microbial communities. Elevated concentrations of Cd, Pb, Zn, and U can suppress microbial abundance and metabolic activity, alter bacterial community structure, and disrupt key biogeochemical cycles. Changes in microbial activity are therefore considered a sensitive indicator of metal and radiation stress in soils of uranium-mining regions [71,72,73].

Taken together, the processes of uptake, accumulation, and biotransformation of heavy metals and uranium in soils of uranium-mining areas determine their pivotal role in the functioning of the “soil–plant–human” system. The long-term retention of contaminants within the soil profile, combined with their incorporation into microbial and plant components of the biological cycle, ensures the persistence of environmental and public health risks well beyond the active mining phase, including the post-operational period. In this context, priority should be given to integrated soil monitoring, assessment of element mobility and bioavailability, and the development of scientifically grounded strategies to mitigate adverse impacts on ecosystems and human health [74,75].

## 5. Heavy Metals in Plants

One of the most informative integrative indicators of the total content, bioavailability, and biological activity of potentially toxic elements in soils is their accumulation in plants. Plant biomass primarily represents the fraction of elements capable of entering the soil solution and participating in biogeochemical cycling, which makes vegetation a sensitive bioindicator of technogenic contamination. The levels of heavy metal accumulation in plants are shaped by the combined influence of biotic and abiotic factors, including species-specific uptake and selectivity, chemical speciation and element mobility, soil type and mineralogical composition, hydrological regime, topography, and climatic conditions. As a result, heavy metal concentrations in plant biomass under natural conditions exhibit pronounced spatial and interspecific variability, a pattern consistently reported across diverse climatic and environmental settings [76].

The accumulation of heavy metals in plants therefore represents a critical nexus between soil geochemistry, plant physiology, and ecosystem health, reflecting not only contamination levels but also the bioavailable fraction of metals within terrestrial ecosystems. This process is governed by a complex interplay of biotic and abiotic factors that determine the bioavailability, uptake, translocation, and ultimate fate of metallic elements within plant tissues [77].

Mining areas create a distinctive environmental setting that functions as a selective “filter” for plant communities. Under increased anthropogenic pressure, some species are eliminated, others undergo degradation or exhibit pronounced morphophysiological alterations, while the most tolerant species adapt and persist. As mining activities intensify and expand to greater depths, the adverse impacts on ecosystems in mining regions increase, particularly due to soil contamination with Pb, Cd, Zn, Cu, and other potentially toxic elements [78,79,80]. Mining operations also result in the transfer of rocks from deep geological horizons to the surface, disrupting the natural distribution of minerals, altering baseline geochemical conditions, and enhancing secondary migration processes of elements [81,82,83,84].

In uranium mining areas, these disturbances are especially pronounced, as elevated concentrations of multiple toxic elements coexist and create heterogeneous contamination patterns that vary significantly across spatial scales. Studies conducted near tailings facilities demonstrate that dominant plant species often exhibit adaptive strategies enabling survival under high metal loads, with accumulation patterns closely linked to soil metal speciation and bioavailability [85].

Numerous studies indicate that soils and vegetation near metal and uranium mining sites are contaminated with varying levels of heavy metals, highlighting the long-term ecological consequences of mining activities for terrestrial ecosystems [86,87,88].

It should be taken into account that some heavy metals are essential micronutrients required by plants in trace amounts for the proper functioning of enzymatic systems, the photosynthetic apparatus, and the regulation of redox processes (e.g., Fe, Zn, Cu, and Mn). However, when their concentrations exceed physiologically optimal levels, these elements become phytotoxic. Moreover, the presence of non-essential metals, such as Cd, Pb, and Hg, which lack biological functions in plants, leads to metabolic disturbances, oxidative stress, and inhibition of plant growth and development [89].

At the cellular level, excessive metal ions may displace essential cofactors from metalloenzymes, bind to sulfhydryl groups in proteins, and induce the formation of reactive oxygen species, resulting in oxidative damage to lipids, proteins, and nucleic acids. Plants respond to such stress through enzymatic and non-enzymatic defense systems, including antioxidant enzymes and metal-chelating compounds, which collectively determine species-specific tolerance to heavy metal exposure [90].

Against this background, a more detailed consideration of the mechanisms of heavy metal toxicity in plants is required to explain observed differences in accumulation, tolerance, and translocation patterns in mining-affected environments. Particular attention should be given to uranium-mining areas, where the combined presence of uranium and associated heavy metals leads to complex, multi-element stress conditions. Understanding these processes is essential for interpreting plant responses at the cellular and physiological levels, assessing element accumulation in vegetation, and evaluating the ecological and sanitary-hygienic implications of contaminant transfer through terrestrial food chains.

## 6. Mechanisms of the Toxic Effects of Heavy Metals on Plants

Excessive concentrations of heavy metals in plant tissues exert adverse effects through both direct and indirect pathways. Direct toxic effects include inhibition of cytoplasmic enzyme activity and damage to cellular membranes and organelles, primarily driven by oxidative stress and the overproduction of reactive oxygen species [91]. Indirect effects include the displacement of essential nutrient elements (e.g., Ca, Mg, Fe, Zn, P) from cation-exchange sites in the root system, disruption of ion transport, and imbalance in plant mineral nutrition [92].

In addition, the suppressive effects of heavy metals on soil microbiota result in declines in the abundance and functional activity of beneficial microorganisms, impairment of organic matter mineralization processes, and reduced soil fertility. Collectively, these effects manifest as decreased photosynthetic activity, inhibited plant growth and biomass accumulation, and, under prolonged exposure, plant mortality [93].

The accumulation of uranium and heavy metals in plants induces a range of physiological disturbances and phytotoxic effects. Vegetation growing on contaminated soils is typically characterized by reduced biomass, decreased rates of biological processes, limited species diversity, and low stress tolerance [94]. The toxicity of heavy metals and uranium manifests in the inhibition of seed germination, retardation of seedling growth, impairment of photosynthesis, disruption of metabolic pathways, and alteration of plant water balance [95].

Soil contamination with heavy metals represents one of the most widespread forms of technogenic pressure on terrestrial ecosystems. Because plants remain in continuous contact with the soil environment, they serve as the primary entry point for heavy metals into terrestrial trophic chains. In this context, the uptake and internal transport of heavy metals within the soil–plant system are multistage processes that involve element acquisition from the soil solution by roots, loading into the xylem, and subsequent translocation to aboveground plant organs [96,97].

The primary sources of soil contamination with heavy metals include industrial emissions and effluents, application of sewage sludge, mining activities, and natural geochemical processes such as volcanism [96]. Once taken up by plants, heavy metals can induce a wide range of physiological disturbances, including stomatal closure, inhibition of photosynthesis, mineral nutrient imbalances, and reduced biomass [96,97]. In response, plants have evolved complex defense mechanisms aimed at limiting metal bioavailability, chelation, detoxification, and the development of systemic tolerance. Symbiotic microorganisms, such as rhizobacteria, endophytes, and phosphate-solubilizing bacteria, play a crucial role in enhancing plant resistance to heavy metals by reducing metal mobility and modulating plant stress responses [98,99].

Typical ranges of phytotoxic concentrations of heavy metals in soils and their corresponding physiological effects on plants are summarized in Table 3. These values represent approximate threshold levels, above which most species exhibit impaired growth, photosynthetic activity, and mineral nutrition.

It should be noted that the threshold concentrations presented in Table 3 primarily reflect the effects of individual heavy metals. Under conditions of technogenic contamination, however, plants are typically exposed to multiple toxic elements simultaneously, which can amplify phytotoxic effects even at concentrations near the established thresholds.

For example, studies on radish seedlings have shown that combined exposure to uranium and cadmium can substantially increase uranium accumulation in both roots and aboveground tissues. Cadmium enhanced uranium phytotoxicity, resulting in reduced biomass, chlorosis, and decreased chlorophyll content. These findings highlight the synergistic risks posed by co-occurring heavy metals in uranium tailings [114].

The data indicate that, under real-world conditions at uranium tailings sites, where contamination is typically multi-element, the phytotoxicity of uranium is influenced not only by its individual effects but also by the presence of co-occurring heavy metals. This underscores the need for a comprehensive approach to assessing the impacts and ecological consequences of contamination on vegetation in uranium-mining regions.

## 7. Accumulation of Uranium and Heavy Metals by Plants in Uranium Mining Areas

According to international studies, in uranium-mining areas, the accumulation of uranium and co-occurring heavy metals in plants can reach levels that pose ecological and public health risks, particularly when contaminated plant biomass enters trophic chains. A defining feature of such regions is that uranium contamination is typically complex, accompanied by the input of a wide range of potentially toxic elements and metalloids (e.g., Th, As, Pb, Mo, Cu, Zn) into soils, collectively forming an anthropogenic geochemical background [115].

Near tailings and processing facilities, these elements are primarily taken up by plants through the rhizosphere, where their concentrations often exceed regional background levels. In this context, uranium, along with heavy metals, actively participates in soil–plant migration processes and can exacerbate the overall toxic burden on plants by interacting synergistically with other elements [116].

The high bioavailability of uranium in the soil–plant system is largely determined by its geochemical behavior. Under aerobic conditions, uranium predominantly occurs in the hexavalent form, U(VI), as the uranyl ion (UO_2_^2+^), which is highly soluble and mobile, facilitating root uptake and translocation to plant tissues [117]. Similar mobility characteristics are observed for several heavy metals, including Cd, Zn, and Cu, promoting their co-accumulation in plants [26,118,119].

Although uranium is not biologically essential, its uptake and accumulation in plants, particularly when combined with heavy metals, have been associated with growth inhibition, reduced photosynthetic activity, and disturbances in mineral nutrition and metabolic processes [120,121]. The co-occurrence of uranium and heavy metals can exacerbate stress effects on plants, leading to chronic physiological responses characterized by long-term disruption of physiological and biochemical functions and redistribution of elements among plant organs.

These mechanisms are supported by field studies conducted in uranium-mining regions, where elevated concentrations of uranium and associated metals in soils are directly reflected in their accumulation in plant tissues. A representative example is the Xiaozhuang uranium mining district in China, where high soil concentrations of uranium and heavy metals are mirrored in plant tissues. In the leaves of dominant tree species (*Sapium discolor*, *Liriodendron chinense*, *Rhus chinensis*, and *Castanopsis carlesii*), uranium concentrations ranged from 0.10 to 0.49 mg/kg, indicating chronic exposure of vegetation under prolonged technogenic influence [70].

Similar patterns of uranium and heavy metal accumulation in vegetation have been observed in other uranium- and metal-mining regions worldwide, highlighting the need to synthesize relevant international data, as summarized in Table 4.

The analysis of the data presented in Table 4 demonstrates pronounced spatial and species-specific variability in the accumulation of uranium and heavy metals by vegetation in uranium- and metal-mining areas. Regardless of site location, uranium and co-occurring metals are consistently taken up by plant roots, as evidenced by elevated element concentrations in plant tissues.

Accumulation levels are strongly influenced by both environmental geochemical characteristics and plant biological traits. For example, in uranium mining regions of China (Xiaozhuang), a clear species-specific pattern of accumulation is observed, with certain tree species exhibiting elevated uranium concentrations in their leaves. In contrast, in the tailings of the Sudetes Mountains (Poland), intensive accumulation of Cd, Zn, and Mn occurs in aboveground plant organs, indicating high bioavailability of these elements in technogenically disturbed soils.

In several plant groups, including grasses and agricultural crops, uranium and heavy metals predominantly accumulate in the root system, with limited translocation to aboveground tissues. This behavior may represent an adaptive mechanism that reduces phytotoxic effects and limits the transfer of contaminants into trophic chains [122]. Conversely, active accumulation of metals in leaves observed in certain species increases the potential ecological and public health risks of contaminated areas.

Overall, these findings confirm the critical role of the plant component in redistributing uranium and heavy metals within the ecosystems of uranium-mining regions and underscore the importance of considering species-specific responses in ecological risk assessment and in the development of phytoremediation and monitoring strategies.

## 8. Uranium and Heavy Metal Accumulation in Vegetation for Human Exposure

The most significant consequence of uranium and heavy metal accumulation by plants is their transfer into food chains and the associated risk to human health. Food products cultivated on contaminated soils may accumulate toxic elements to levels that pose a potential hazard for human consumption. Food-chain studies conducted in uranium mining regions of northwestern New Mexico (Navajo Nation) have demonstrated that uranium and associated heavy metals accumulate in locally harvested plants and forage and are subsequently detected in sheep tissues, particularly in the liver and kidneys, confirming plant-mediated transmission of contaminants along the food chain from contaminated soils and water to human dietary intake [136]. Similar food-chain transfer processes have been reported in other uranium mining regions, where uranium-associated radionuclides and heavy metals were detected in tissues of large herbivores (e.g., moose and cattle), confirming trophic transfer of contaminants and supporting vegetation-mediated exposure pathways for humans [137]. Combined contamination involving uranium and associated heavy metals increases the likelihood of both chemical and radiological exposure to the human body through food and drinking water [95,120,138].

The intake of heavy metals and uranium into the human body may cause a wide range of adverse effects, including neurological disorders, damage to the kidneys and liver, dysfunction of the cardiovascular system, and an increased risk of cancer [113,120,139]. Under conditions of water scarcity, where wastewater and contaminated sources are used for irrigation, the risk of biotransfer of toxic elements from soil to plants and subsequently to humans increases, necessitating enhanced monitoring and risk assessment [139,140]. Quantitative evaluation of potential health impacts in such systems is commonly performed using the hazard quotient (HQ), hazard index (HI), and carcinogenic risk (CR) indicators, which allow assessment of multi-component exposure via different pathways [138,140]. HQ and HI values exceeding the threshold of 1 indicate an increased probability of chronic adverse health effects, particularly among vulnerable population groups, including children and individuals with compromised health [138].

Uranium, as a chemically toxic and radiologically active element, represents an additional source of hazard. Its bioavailability and accumulation in plants depend on soil geochemical conditions, such as pH, redox potential, and microbiological activity, which control uranium speciation and the rate of root uptake [120]. The combined presence of uranium and heavy metals in soils increases the overall toxic burden, reflected in higher calculated HQ and HI values for deterministic human exposure pathways.

Thus, quantitative risk indicators (HQ, HI, and CR) provide an objective basis for assessing the sanitary and hygienic implications of uranium and heavy metal accumulation in vegetation, identifying vulnerable population groups, and developing targeted measures for health risk management and environmental protection [138,140].

## 9. Discussions

The analysis of published data demonstrates that uranium mining creates a distinctive geochemical environment in which uranium is released together with a broad suite of heavy metals, forming complex, multi-element contamination patterns that persist long after mining activities cease. Across different geographical regions, tailings, waste rock, and disturbed ore-bearing strata consistently function as long-term sources of potentially toxic elements. Their redistribution through leaching, acid mine drainage, and atmospheric dispersion establishes hydrologically driven, spatially and temporally persistent migration pathways linking soils, surface and groundwater, and vegetation. This confirms that uranium-mining landscapes operate as integrated systems in which contamination cannot be assessed in a single environmental compartment without considering the others.

Water bodies primarily serve as transport media, enabling contamination to spread beyond mining sites. The reviewed case studies show that dissolved and particle-bound metals frequently exceed regulatory thresholds, particularly under acidic and oxidizing conditions that enhance metal solubility. These findings are consistent with the dominant role of hydrogeochemical controls, particularly pH, redox potential, and dissolved organic matter, in determining metal mobility. At the same time, the reviewed data indicate that similar hydrogeochemical drivers may result in markedly different contamination outcomes depending on local lithology, mineralogical composition of wastes, and the legacy of applied mining technologies. However, regional differences in lithology and mining technology strongly influence the nature of pollution, which explains why some areas show extreme exceedances of drinking water quality standards (including WHO guideline values and national standards), while others experience moderate but chronic pollution.

Soils represent the main long-term sink for uranium and heavy metals and therefore play a central role in shaping ecological risks. The persistence of metals over decades to millennia highlights the limited self-purification capacity of contaminated soils, including natural attenuation and biogeochemical immobilization processes. High spatial heterogeneity of metal distributions reflects both natural background variability and the intensity of technogenic inputs. Importantly, soil does not merely store contaminants but actively regulates their bioavailability through sorption, complexation, and redox-driven transformations. This regulatory function links abiotic contamination to biological exposure pathways.

Vegetation reflects the bioavailable fraction of metals and serves as an indicator of contamination through bioaccumulation in plant tissues. Species-specific accumulation patterns observed in different uranium-mining regions indicate that plant physiological traits and root–soil interactions strongly influence uptake and translocation. While some species restrict metals mainly to roots, others accumulate them in aboveground tissues, increasing the probability of entry into food webs. The reviewed data also show that combined exposure to uranium and heavy metals can produce synergistic phytotoxic effects, even when individual elements occur near threshold levels. This interaction is particularly relevant for uranium-mining areas, where multi-element contamination is the norm rather than the exception.

From an ecological perspective, chronic metal exposure alters soil microbial communities, reduces plant diversity, and impairs ecosystem functioning. From a sanitary-hygienic standpoint, the soil–water–plant transfer chain represents a critical pathway for human exposure. Risk indicators such as HQ, HI, and CR provide a quantitative framework for evaluating cumulative impacts, especially in regions where contaminated water and locally grown food are used. The evidence reviewed here indicates that uranium and its associated heavy metals represent concomitant chemical and radiological stress factors, which underscores the need to take these factors into account comprehensively when assessing environmental risk.

An important implication of the reviewed studies is that the environmental impact of uranium mining cannot be reliably interpreted using single-element or single-medium assessments. Although many investigations focus on total concentrations of individual metals in water or soil, the evidence summarized in this review indicates that bioavailability-controlled processes and interactions among soils, waters, and vegetation largely determine contaminant behavior in mining-affected ecosystems. As a result, comparable concentration levels may correspond to substantially different ecological outcomes depending on local geochemical conditions and biological responses.

Another key aspect is the predominance of multi-element contamination as a defining feature of uranium-mining environments. Uranium is rarely present in isolation and is consistently associated with a suite of heavy metals whose combined occurrence may amplify ecological effects. Such mixed-contamination scenarios complicate the interpretation of field data, as ecosystem responses cannot be inferred solely from individual element concentrations. This helps explain the wide variability in reported impacts among sites with apparently similar levels of uranium or associated metals.

In addition, considerable variability among published studies reflects differences not only in environmental settings but also in sampling design, analytical techniques, and reporting metrics. Variations in metal speciation, redox conditions, and organic matter content may lead to contrasting conclusions regarding metal mobility and accumulation even within similar mining contexts. These inconsistencies highlight the importance of interpreting reported concentrations within their specific geochemical and ecological framework rather than relying exclusively on generalized threshold values.

Overall, the findings emphasize the necessity of ecosystem-based approaches in uranium-mining regions, integrating soil, water, and vegetation data with geochemical and biological indicators. This integrated perspective is essential for understanding coupled contamination processes and for supporting scientifically grounded strategies aimed at mitigating long-term ecological and public health risks associated with uranium mining.

In this context, increasing attention has been directed toward the development of restoration and land-reclamation strategies aimed at reducing the long-term environmental impacts of uranium mining. These approaches include physical stabilization of tailings and waste rock, chemical immobilization of contaminants, and the application of phytoremediation techniques using metal-tolerant plant species. Vegetation-based restoration, in particular, plays a dual role by limiting erosion and contaminant dispersion while simultaneously influencing metal bioavailability through rhizosphere processes. However, the effectiveness of these measures is strongly dependent on site-specific geochemical conditions, including pH, redox potential, and mineral composition, as well as on the selection of appropriate plant species. Therefore, successful mitigation requires integrated, site-adapted reclamation strategies that combine engineering, geochemical, and biological approaches.

## 10. Conclusions

The analysis of published data indicates that uranium mining activities lead to long-term contamination of environmental compartments, primarily soils and surface and groundwater, which serve as the primary sources of uranium and heavy metal migration within affected ecosystems. Elevated concentrations of uranium and associated potentially toxic elements in soils and water bodies create conditions for their active transfer into the biological cycle.

Soils act as the main accumulation medium for uranium and heavy metals, while water facilitates their mobility and redistribution across the landscape. Changes in physicochemical properties of soils and waters, including pH, redox conditions, and dissolved organic matter, significantly influence the bioavailability of these elements and determine their uptake by plants.

Vegetation represents a key link in the migration chain connecting abiotic components (soil and water) with biotic systems and human exposure pathways. The reviewed studies demonstrate that plants can accumulate uranium and heavy metals at levels reflecting both total and bioavailable fractions in soils and waters, with pronounced species-specific differences in uptake and translocation.

From an ecological and public health perspective, the transfer of uranium and heavy metals through the “soil–water–plant–human” system poses a significant long-term risk, particularly in arid and semi-arid regions where contaminated water and plant resources are actively used. Quantitative health risk assessment using hazard quotient (HQ), hazard index (HI), and carcinogenic risk (CR) calculations is essential for evaluating cumulative exposure and identifying vulnerable population groups.

The findings highlight the need for an integrated environmental assessment approach in uranium mining regions that combines analyses of soil, water, and vegetation. Such an approach is critical for effective environmental monitoring, risk management, and the development of remediation strategies, including regulating water use, rehabilitating soils, and the controlled application of phytoremediation technologies.

## Figures and Tables

**Table 1:** Major pathways of heavy metal migration in uranium-mining areas (compiled based on literature data).

System Component	Migration Process	Key Controlling Factors	Typical Elements	Environmental and Sanitary Implications	References
**Soil → Water**	Leaching from contaminated soils and mining wastes	pH, Eh, mineralogical composition, precipitation, hydrodynamics	U, Cd, Pb, Cr, Zn, Ni	Contamination of surface and groundwater; secondary pollution	[31,32,33,34,35,36]
**Soil → Vegetation**	Root uptake and accumulation	Metal bioavailability, organic matter, and plant species	Cd, Pb, Cr, Cu, Ni, Zn, Mn, U	Phytotoxicity; entry into food chains; human health risks	[27,37,38,39,40,41]
**Water → Vegetation**	Uptake from irrigation and surface water	Metal concentration, chemical speciation, and irrigation regime	Cd, Pb, Zn, Cu, Ni	Accumulation in crops; food safety risks	[27,42]
**Soil ↔ Sediments**	Sedimentation and secondary remobilization	pH, Eh, organic matter content, microbial activity	Cd, Pb, Cr, Zn, U	Formation of secondary contamination sources	[34,36,39]
**Tailings and waste dumps → Soil and water**	Aerosol dispersion; leaching	Precipitation, grain size, waste isolation degree	U, Pb, Zn, As, Cd, Tl	Long-term contamination of ecosystems	[37,39,43]

**Table 2:** Heavy metal contamination of water resources in uranium-mining areas worldwide.

Region (Country)	Uranium-Mining Site/Area	Main Contaminants	Environmental Compartment	Concentration Ranges	Key Findings	Reference
**Africa**						
Tanzania	Proposed Mkuju River uranium project	Al, Mn, Fe, Pb	Surface water, groundwater	Al: up to 9049 µg/L; Mn: up to 1452 µg/L; Fe: up to 53,890 µg/L; Pb: up to 78.68 µg/L	Elevated concentrations of Al, Mn, Fe and Pb at selected sites exceeded international water quality guidelines; other metals (Cr, Ni, Zn, Cu, Cd, Co, As, Th, U) remained below threshold values	[48]
Namibia	Langer Heinrich uranium mine	U, V, Li	Groundwater	U: 80–91,000 µg/L; V: 20–118,000 µg/L; Li: 40–410 µg/L	Elevated metal concentrations due to seepage from uranium tailings	[49]
**Asia**						
China	Xiangshan uranium mining area (Jiangxi Province, Fuhe River basin)	U, Th, Cd, Cr, Pb	Surface water, bottom sediments	Surface water: U 3.33 µg/L, Th 2.23 µg/L; Sediments: Cd 1.74 mg/kg, Cr 259.8 mg/kg, U 8.04 mg/kg	Concentrations exceeded regional background levels by 4.9–24.7 times; pollution indices (CF, Igeo, RI) indicated high ecological risk, mainly from Cd, Cr and U	[19]
India	Bagjata and Bhaduya uranium mines (Jharkhand)	Fe, Mn, Zn, Pb, Cu, Ni	Groundwater	Bagjata: Fe 3250–5080 µg/L, Mn 130–340 µg/L; Bhaduya: Fe 60–150 µg/L, Mn 100–190 µg/L	Most metals were within permissible limits; Fe and Mn exceeded drinking water standards at several sampling points	[50]
**North America**						
USA	Navajo Nation legacy uranium-mining areas	V, As, Mn, U, Li, Ca	Unregulated surface and groundwater sources	V: up to 520 µg/L; Mn: up to 14,700 µg/L; U: up to 490 µg/L; As: up to 190 µg/L	Periodic exceedance of U.S. drinking water standards; elevated levels of V, Mn, As, Li and U persisted decades after mining cessation	[51]
USA	Juniper uranium mine (California, abandoned)	U, Mn, Fe, As, Zn, Cu, Ni	Surface water	U: up to 52.4 µg/L; Mn: up to 243.8 µg/L; Fe: up to 5550 µg/L; As: up to 1.6 µg/L; Zn: up to 13.1 µg/L	Elevated U near source, decreasing downstream; transport controlled by dilution and sediment adsorption.	[52]
Canada	Uranium mining area (Saskatchewan)	Se	Surface water	Se: 0.1–2.7 µg/L	Low Se in water but significant bioaccumulation in plankton, invertebrates and fish	[53]
**South America**						
Brazil	Caldas uranium mining area (Minas Gerais State)	U, Mn, Zn	Surface water	U up to 5620 µg/L; Mn up to 75,000 µg/L; Zn up to 18,600 µg/L	U, Mn, Zn concentrations exceeded guideline values in surface waters affected by uranium ore mining	[54]
**Australia**						
Australia	Ranger uranium mine (post-closure)	U, Mn, Mg	Groundwater	U up to 0.7 µg/L; Mn up to 350 µg/L; Mg up to 350,000 µg/L	Mixture toxicity was observed, with Mn and Mg identified as the dominant contributors to ecological risk, while uranium played a minor role due to low concentrations	[55]
Australia	Rum Jungle uranium- copper mine	Cu, Zn, Mn, Ni, U	Surface water, groundwater	Cu: up to 1100 µg/L; Mn: up to 2000 µg/L; Zn: up to 670 µg/L; U: up to 63 µg/L	Acid mine drainage causes long-term heavy metal contamination of surface and groundwater, persisting after mine rehabilitation	[56]
**Europe**						
Germany	Former Wismut uranium mining district (Saxony)	U, Pb	Groundwater	U up to 380 µg/L; Pb up to 120 µg/L	Post-mining monitoring reveals persistently elevated U and Pb concentrations in groundwater, indicating long-term contamination of river systems associated with historical uranium mining	[57]
Portugal	Cunha Baixa abandoned uranium mine (Central Portugal)	U, Al, Mn, Ni, Zn	Groundwater	U: 595–5700 µg/L; Mn: 100–31,900 µg/L; Al: 130–57,200 µg/L; Ni: 29–626 µg/L; Zn: 134–3288 µg/L	Groundwater is affected by acid mine drainage, characterized by low pH and elevated U, Al, and Mn concentrations, posing risks to irrigation and human health	[58]
Romania	Ciudanovita and Lisava (former uranium mining and tailing dump sites, Banat region)	Cd, Pb, Cu, Zn (also Al, Cr, Mn, Fe, Ni, Co, Sr)	Groundwater and surface water	Groundwater: Cd: 0.15–28.43 µg/L; Pb: 3.62–162.33 µg/L; Cu: 0.94–141.19 µg/L; Zn: 7.89–67.96 µg/L Surface water: Cd: 0.01–16.71 µg/L; Pb: 1.25–28.02 µg/L; Cu: 0.53–44.49 µg/L Zn: 14.39–75.54 µg/L	Elevated heavy metal concentrations in both groundwater and surface water due to legacy uranium mining and tailing dumps	[59]
Spain	Los Ratones uranium mine	U, Fe, As	Groundwater	U: 1–104 µg/L	Elevated uranium concentrations near mining structures; sulfide oxidation enhances metal release, while iron oxyhydroxides act as sinks controlling U and As mobility	[60]

Note: Water concentrations are expressed in µg/L (values originally reported in mg/L were converted), while bottom sediment concentrations are expressed in µg/kg.

**Table 3:** Main physiological effects and morphological manifestations of the toxic effects of heavy metals on plants.

Heavy Metal	Main Physiological Effects	External Symptoms	References
**Pb**	Inhibition of seed germination, inhibition of protease and amylase activity, inhibition of photosynthesis, and induction of oxidative stress	Chlorosis, growth inhibition, root and shoot deformity	[100,101]
**Cd**	Impaired CO_2_ fixation, nitrate reductase inhibition, inhibition of Ca, Mg, Fe transport, decreased ATPase activity of membranes	Chlorosis, growth inhibition, root end browning, root and shoot length reduction, biomass reduction, plant death at high exposure levels	[102,103,104]
**Zn**	Inhibition of photosynthesis, impaired enzymatic activity, induction of oxidative stress, induced deficiency of Fe, Mn, Cu and P, impaired transport of trace elements from roots to shoots	Chlorosis of young leaves with subsequent spread to old leaves, purple color of tissues, growth retardation, inhibition of the development of root and aboveground biomass, accelerated plant aging	[104,105,106]
**Cr**	Induction of oxidative stress and lipid peroxidation, cell membrane damage, degradation of photosynthetic pigments, disruption of chloroplast ultrastructure, inhibition of CO_2_ fixation, electronic transport and photophosphorylation, suppression of amylase activity and alteration of protease activity, disruption of metabolism and induction of antioxidant systems (SOD, catalase), synthesis of phytochelatins and other protective metabolites	Reducing seed germination, inhibiting root and shoot growth, reducing biomass, chlorosis, delaying plant development	[107,108,109,110]
**Ni**	Disturbance of mineral nutrition, imbalance of ion homeostasis, change in lipid composition, and decrease in H^+^—ATPase activity of the plasma membrane, inhibition of photosynthesis and fixation of CO_2_ (suppression of enzymes of the Calvin cycle), decrease in chlorophyll and stomatal conduction, violation of water balance, and decrease in water holding capacity of tissues	Chlorosis and necrosis of leaves, inhibition of root and shoot growth, reduced yield, reduced biomass, and violation of the water regime of plants	[111,112,113]

**Table 4:** Accumulation of uranium and related elements in plants of uranium mining regions (according to literature data).

Region/Site	Object Type	Plant Types/ Vegetation	Elements	Concentrations in Plants	Key Findings	Reference
**Asia**						
Xiaozhuang, China	Uranium ore deposit	*Sapium discolor*, *Liriodendron chinense*, *Rhus chinensis*, *Castanopsis carlesii*	U, Mn, Co, Cd, Cu, Zn	U in leaves: 0.10–0.49 mg/kg	Pronounced species specificity of accumulation	[70]
Dabaoshan, Guangdong, China	Polymetallic mine	*Miscanthus floridulus*	Pb, Zn, Cu, Cd	Zn 71–195; Pb 9–160; Cu 10–83; Cd 0.26–30 mg/kg (root > stem > leaf)	Root accumulation, limited translocation into leaves; Zn dominates	[122]
Tummalapalle, India	Uranium mining area	*Vegetable* *crops*	Cu, Fe, Zn, Pb, Mn, Mo	Cu: 0.03–4.65 mg/kg; Fe: 6.29–62.44 mg/kg; Zn: 0.16–1.12 mg/kg; Pb: 0.01–0.61 mg/kg	Elevated heavy metals in vegetable crops	[123]
Aktau, Kazakhstan	Uranium mining area	*Artemisia* *austriaca*	U	U in leaves: 0.465–1.209 mg/kg; in roots: 0.674–1.954 mg/kg	Elevated U in plants; accumulation linked to dust transport; spatial variability observed	[124]
Transbaikalia, Russia	Uranium mining and tailings dumps	*Agropyron cristatum*, *Leymus chinensis*, *Stipa baicalensis*	U, Pb, Zn, Cu, Mn, As	U up to 21.4; Pb up to 47.7; Zn up to 750; Cu up to 155; Mn up to 594; As up to 27.7 mg/kg	Herbaceous steppe grasses exhibit a strong accumulation of U and heavy metals under the impact of uranium mining	[125]
**Africa**						
Manyoni, Tanzania	Uranium mining area	*Leafy* *vegetables* *(pea, pumpkin, spinach)*	Ni, Cu, Zn, Fe, Pb	Ni: up to 67.3 mg/kg; Cu: up to 14.9 mg/kg; Zn: up to 79.8 mg/kg; Fe: up to 478.6 mg/kg; Pb: up to 1.6 mg/kg	Elevated heavy metals in edible plants; Ni and Pb exceed safety limits; potential food chain contamination	[126]
Lolodorf (Mvengue, Awanda), Cameroon	Uranium-bearing area	*Leafy vegetables and tuber crops*	Al, Cr, Mn, Th, U, Fe, Ni, Cu, Zn	Al: 671.1 mg/kg; Cr: 3.1 mg/kg; Mn: 117.1 mg/kg; Th: 0.2 mg/kg; U: 2.9 mg/kg; Fe: 292.9 mg/kg; Ni: 9.2 mg/kg; Cu: 11.9 mg/kg; Zn: 43.1 mg/kg	Elevated trace metals (Al, Cr, Fe) exceeding WHO/FAO limits; potential non-carcinogenic and carcinogenic risks identified	[127]
**Australia**						
Mary Kathleen, Australia	Uranium mining area	*Aerva javanica*, *Cymbopogon bombycinus*, *Aristida longicollis*, *Acacia chisholmii*	U, Th, Cu, Pb, Zn, Ni, As	U: up to 318 mg/kg; Cu: up to 571 mg/kg; Zn: up to 706 mg/kg	Elevated U and heavy metals; higher accumulation in contaminated sites; species-dependent uptake	[128]
**Europe**						
Sudetenland Mountains, Poland	Uranium mining waste dumps	*Salix caprea*, *Betula pendula*, *Rubus idaeus*	Cd, Zn, Pb, Mn	Cd up to 24.7; Zn up to 386; Pb up to 27.9; Mn up to 1724 mg/kg in leaves	High bioavailability of Cd and Zn	[129]
Wismut region, Germany	Uranium mining area	*Triticum* *aestivum*	Cd, Cu, Zn, Pb, U, As	Cd: up to 29.4 mg/kg (roots); Zn: up to 1203 mg/kg; U: up to 8.49 mg/kg	Preferential accumulation in vegetative tissues with restricted translocation to grains	[130]
Rozna-Olsi, Czech Republic	Uranium mining area	*Alnus glutinosa*, *Acer platanoides*, *Salix fragilis*, *Corylus avellana*, *Aesculus hippocastanum*	U, Cd, Cu, Pb, Zn	U in leaves: 0.1–51.8 mg/kg; Cu: 9.5–118 mg/kg; Pb: 1.8–326 mg/kg; Zn: 34–144 mg/kg; Cd: 0.12–2.14 mg/kg	High variability in accumulation; Cu frequently exceeded toxicity limits; elevated U content; no clear decrease with distance from the source.	[131]
**North America**						
Panama, Central America	Abandoned gold mine tailings	*Oryza sativa*, *Zea mays*, *Manihot esculenta*	As, Sb, Cu, Zn	As up to 54.5; Sb up to 9.7; Cu up to 57.3; Zn up to 5.7–273.1 mg/kg;	Wide variability of bioaccumulation (weak to strong)	[132]
North America	Uranium-contaminated soils (experiment)	*Helianthus annuus*, *Brassica juncea*	U	Roots up to 6200 mg/kg, aboveground organs (shoots up to 102 mg/kg)	Preferential accumulation in the root system. Promising species for phytoremediation	[133]
Sonoran Desert, North America	Abandoned uranium mine	*Larrea tridentata*, *Encelia farinosa*	U, As, Cd, Zn, Cu	U up to 20; As 10–122; Cd 6.4–14.9; Zn 4.1–28.1; Cu 3.0–15.6 mg/kg	Low–moderate U, As, Cd, Zn accumulation	[134]
Navajo Nation, North America	Uranium mining impacted area	*Thelesperma megapotamicum*	U, Cd, Pb, As, Mo, V	U: 0.019–0.114 mg/kg; Cd: 0.35 mg/kg; Pb: 0.30–0.80 mg/kg	Higher accumulation in roots; Cd exceeded WHO limits; potential human exposure risk	[135]
**South America**						
Brazil	Legacy uranium mining area	*Zea mays*	U, As, Cd, Pb, Zn	U: 0.01–0.3 mg/kg; Cd: 0.23–24 mg/kg; Zn: 10–52 mg/kg; Pb: 0.01–1.04 mg/kg	Bioaccumulation in crops; food chain risk	[8]

Note: All concentrations are expressed in mg/kg as reported or converted from original units.

## Data Availability

The data presented in this study are available upon request from the corresponding authors, Madina Kairullova and Danara Ibrayeva.
